# Landscape Evolution and Simulation of Rural Settlements around Wetland Park Based on MCCA Model and Landscape Theory: A Case Study of Chaohu Peninsula, China

**DOI:** 10.3390/ijerph182413285

**Published:** 2021-12-16

**Authors:** Xin Fan, Wenxu Luo, Haoran Yu, Yuejing Rong, Xinchen Gu, Yanjun Zheng, Shengya Ou, Damien Sinonmatohou Tiando, Qiang Zhang, Guiling Tang, Jiangfeng Li

**Affiliations:** 1 School of Public Administration, China University of Geosciences, Wuhan 430074, China; fanfx8@edu.cug.cn (X.F.); damientiando90@gmail.com (D.S.T.); jfli0524@163.com (J.L.); 2 State Key Laboratory of Earth Surface Processes and Resource Ecology, Beijing Normal University, Beijing 100875, China; 3 International Education College, China University of Geosciences, Wuhan 430074, China; intlstudents@cug.edu.cn (W.L.); tangguiling@cug.edu.cn (G.T.); 4 School of Architecture and Planning, Anhui Jianzhu University, Hefei 230022, China; yuhaoran2021@ahjzu.edu.cn (H.Y.); zhangqiang1023@foxmail.com (Q.Z.); 5 Anhui Urbanization Development Research Center, Anhui Jianzhu University, Hefei 230022, China; 6 State Key Laboratory of Urban and Regional Ecology, Research Center for Eco-Environmental Sciences, Chinese Academy of Sciences, Beijing 100085, China; 7 University of Chinese Academy of Sciences, Beijing 100049, China; 8 State Key Laboratory of Hydraulic Engineering Simulation and Safety, School of Civil Engineering, Tianjin University, Tianjin 300072, China; 9 State Key Laboratory of Simulation and Regulation of Water Cycle in River Basin, China Institute of Water Resources and Hydropower Research, Beijing 100044, China; 10 Student Innovation & Entrepreneurship Guidance Centre, China University of Geosciences, Wuhan 430074, China; Zhengyanjun@cug.edu.cn; 11 School of Geography and Tourism, Shaanxi Normal University, Xi’an 710119, China; osy201705598@snnu.edu.cn

**Keywords:** land use change, rural settlements, MCCA model, spatial pattern evolution, peri-urban areas, urban-lake symbiosis

## Abstract

As a transitional zone between urban and rural areas, the peri-urban areas are the areas with the most intense urban expansion and the most frequent spatial reconfiguration, and in this context, it is particularly important to reveal the evolution pattern of rural settlements in the peri-urban areas to provide reference for the rearrangement of rural settlements. The study takes five townships in the urban suburbs, and explores the scale, shape, spatial layout, and spatial characteristics of the urban suburbs of Hefei from 1980 to 2030 under the influence of urban-lake symbiosis based on spatial mathematical analysis and geographical simulation software. The study shows that: (1) the overall layout of rural settlements in the study area is randomly distributed due to the hilly terrain, but in small areas there is a high and low clustering phenomenon, and the spatial density shows the distribution characteristics of “high in the east and low in the west”; (2) since the reform and opening up, there are large spatial differences in the scale of rural settlements in the study area. (3) Different development scenarios have a strong impact on the future spatial pattern of rural settlement land use within the study area, which is a strong reflection of policy.

## 1. Introduction

The evolution of rural settlements is a result of the interaction between humans and the natural environment, and a vehicle for the long-term development activities of human society [1,2,3]. The emergence, development, stabilization, and migration patterns of rural settlements are characterized by distinct phases [4], with different response time periods. Since the economic reforms of the late 1970s, rural settlements in China have been undergoing unprecedented transformations [5,6], which have both spatial and geographical dimensions and are closely linked to China’s political and economic transformation. In China, with a long history of agriculture [7] and a large rural population, agricultural development has brought about more complex and diverse problems than in other countries [8,9]. Since the reform and opening up in 1978, China has given high priority to urban development but has neglected rural development, which has led to a slow and relatively uneven development in most rural areas [10,11,12]. To address this problem, the Chinese government has initiated a number of strategies, including the transformation of rural and urban development [13], a new type of urbanization and a rural revitalization strategy [14,15,16,17]. However, the vastness of China’s territory and the complex and diverse problems that need to be faced hinder the effective implementation of such policies. It is therefore extremely important to analyse the evolution and development process of regional rural settlements [18,19,20,21] in order to predict the effects of various policy implementations. These studies can serve as a reference for the effective spatial planning of rural settlements in the context of new urbanization and rural revitalization policies.

The formulation of effective planning and development strategies for rural settlements depends on a detailed analysis of the spatial evolution patterns of rural settlements [22,23,24,25]. Studies on the evolution of rural settlements have been conducted in China and abroad since the early 20th century [26,27], focusing on qualitative descriptions of the formation, development, and distribution patterns of rural settlements and the relationship between rural settlements and their geographical environment [28,29]. Until the late 1990s, research on the evolution of rural settlements focused mainly on the underlying mechanisms of spatial and temporal evolution and development of rural settlements, although some scholars also paid some attention to the influence of social, economic, and political institutions [30]. Among these, the evolutionary patterns and transformation mechanisms of the rural settlements around them have attracted widespread attention worldwide due to the negative effects of urbanization. In China, in particular, the accelerated urbanization process and the corresponding migration of farmers to cities have led to a series of problems, prompting research on the development of rural settlements, the transformation of their functions, and the changes in their industrial structure [31]. The results are shown as follows. The spatial differentiation of rural settlements is obvious in Central China, and the “plain–hill–mountain” regional gradient, “developed–undeveloped–underdeveloped” economic gradient, and “suburb–outer suburb–country” distance gradient are prominent. [32] It is worth noting that, despite the diversity of previous studies on the evolution of rural settlements, most of them have focused mainly on the development of rural settlements in the two decades since the 21st century, with little analysis of the evolution of rural settlements over a longer period of time [33], and the suitability of policy implementation for the future development of rural settlements requires a deeper understanding based on the long-term evolution of rural settlements.

Simulating future land use changes by testing different scenarios under relevant policies [34,35,36] is an effective means of predicting the effects of policy implementation. At present, scholars at home and abroad have conducted relevant studies based on different development scenarios using the PLUS model [37], FLUS model [38], and CLUS model [39,40,41], such as comparing land use planning policies before and after implementation, the simulation of different ecological protection measures strategies to explore different environmental problems caused by land use changes, and the prediction of land use change trends in different socio-economic development contexts. Liang et al. proposed a mixed cell-based CA model (MCCA). The cell of the model contains the coverage ratio of various types of land, that is, the cell contains the land use structure, which is the spatiotemporal dynamics of the mixed land use structure. Modeling provides a new perspective [42]. Most of these studies have dealt with land use change, but rarely have they modelled or predicted the development trends of rural settlements [38,39]. Therefore, with the help of various scenario settings, the evolution of rural settlements under different policy interventions can be modelled and predicted, which is crucial for assessing the future development of rural settlements and identifying appropriate development patterns.

The developed coastal regions of the Southeast and the provincial capitals are the most typical urbanised regions in China, and the rapid urbanisation process has affected the development of rural settlements in the suburbs of the cities, causing them to exhibit unique characteristics [40,41,42]. In-depth research on the changing patterns of rural settlements in rapidly urbanising areas and the driving forces behind the changes can help to accurately define the problems of regional land use, and thus guide management decisions on rural land use [43].

In 2017, the country’s first national tourism and recreation zone was listed, and the establishment of the National Tourism Demonstration Zone around Chaohu Lake has affected not only the development of Hefei city, but also the production and lifestyle of rural settlements within it. Chaohu Peninsula is the core area of the National Tourism Demonstration Zone around Chaohu Lake, and because of its special geographical location between Hefei city and Chaohu city, its rural settlements are subject to multiple factors, and it is a representative area for exploring the development of rural settlements in the coexistence state of city and lake.

To understand the development trend of rural settlements under the urban-lake symbiosis development model and to compensate for the lack of rural settlement evolution in past studies, this paper focuses on (1) analysing the characteristics and drivers of rural settlement evolution in the Chaohu Peninsula since the reform and opening up, and understanding the spatial and temporal evolution patterns of rural settlements under the influence of complex location and policies. (2) Moreover, the MCCA (mixed-cell cellular automata) model was used to simulate the evolution of rural settlements on the Chaohu Peninsula in 2030 to compare the development characteristics of rural settlements under different policy scenarios. (3) The evolution characteristics of rural settlements were analysed under different development scenarios, with the aim of providing a reference for the future development direction of rural settlements on the Chaohu Peninsula.

## 2. Materials and Methods

### 2.1. Study Site

**Figure 1:**
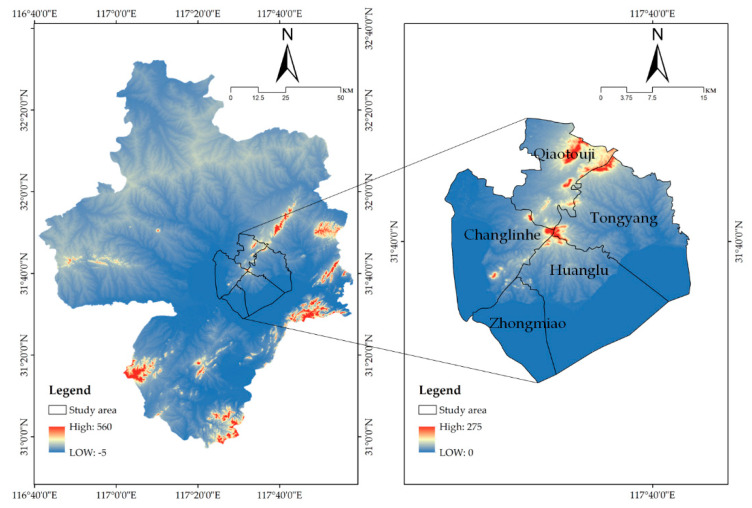
Geographical location and topography of the study area.

The Chaohu Peninsula includes all the administrative areas of Zhongmiao Street, Huanglu Town and Jiong Yang Town in Chaohu City, as well as Changlinhe Town and Qiaotouji Town in Feidong County, with a total land area of 460 square kilometres. It is 3.5 km from downtown Hefei and 13 km from downtown Chaohu. The topography in the territory is the transition zone from the Jianghuai hills to the Yangtze River Plain. The topography is relatively complex, divided into five types of landforms: low mountains, hills, hills, plains (lakeside plains and wavy plains), and waters (Figure 1).

### 2.2. Data Source

The research data mainly includes: (1) Land use data with a spatial resolution of 30 m for the study area in 1980, 1990, 2000, 2010, 2015, and 2020, obtained from the Resource and Environment Information Centre of the Chinese Academy of Sciences, based on Land TM and Landsat-8 remote sensing data as the main data source through manual visual interpretation, with an accuracy of 91.49% for the study area; (2) 30 m precision DEM data, meteorological data, and the 2020 study area zoning and road data were obtained from the geospatial data cloud platform; (4) socio-economic data and planning texts were obtained from government departments. All data were processed through the ARCGIS 10.6 platform, and all data were unified with the coordinate system GCS_Krasovsky_1940 and an accuracy of 30 m.

### 2.3. Research Framework

**Figure 2:**
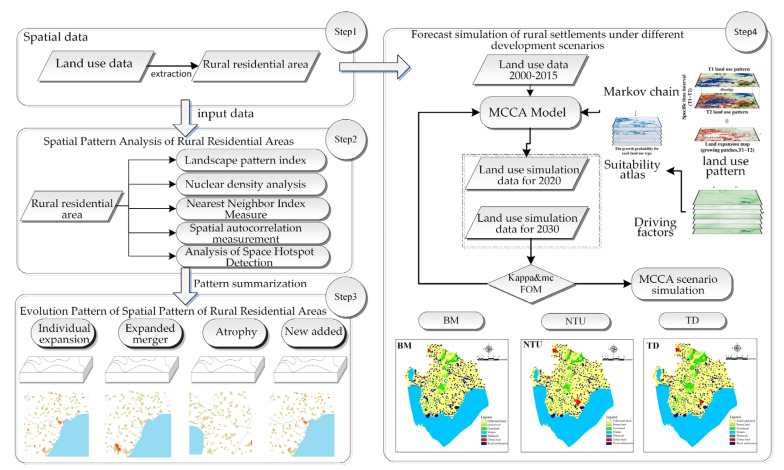
Research Technology Roadmap.

The historical spatial data of rural settlements in the Chaohu Peninsula were extracted by processing land use data for five periods. The spatial pattern of rural settlements in the Chaohu Peninsula in different periods and the corresponding evolutionary characteristics were determined through the measurement of landscape pattern changes, nuclear density, nearest neighbour index, and spatial autocorrelation in the study area. The MCCA model was then used to simulate the land use of rural settlements in the Chaohu Peninsula under different policy development contexts, and the technical route is shown in Figure 2.

### 2.4. Analysis of the Spatial Pattern of Rural Settlements

#### 2.4.1. Landscape Pattern Index

Landscape indices can provide a high overview of information on landscape patterns, simple quantitative indicators that respond to certain aspects of their structural composition and spatial configuration characteristics. In this paper, we use landscape pattern indices to describe the changing characteristics of rural inhabitants in different periods of time, and select representative indices to analyse changes in the landscape patterns of rural settlements in the study area according to relevant studies [41,42]. There are six indices: patch area (CA), number of patches (NP), maximum patch index (LPI), mean patch area (MPS), landscape shape index (LSI), and mean patch shape index (Shape_MN). The characteristics and meanings of each landscape index are shown in Table 1, and each index was calculated using Fragstats 4.2 software.

#### 2.4.2. Kernel Density Analysis and Nearest Neighbour Index Measures

Using the kernel density estimation method to reflect the spatial distribution characteristics and changing trends of rural settlements, the higher the kernel density value of the point being estimated, the larger the estimated kernel density around it; conversely, the smaller it is [22]. Its formula is:

(1) $$\lambda \left(s\right)=k\overset{n}{\underset{i=1}{\sum }}\frac{1}{\pi r^{2}}\left\{\frac{d_{is}}{r}\right\}$$

where *λ(s)* is the kernel density of a planar location country cluster(*s*), *r* is the search radius, *d_is_* is the distance from point *i* to point *s*, and *k* is the weight function of the kernel.

The average nearest neighbour index is the distance between the minimum distance between the points analysed and the ideal nearest neighbour, expressing certain characteristics of the spatial distribution of points. In this study area, it is mainly used to determine the overall distribution pattern of rural settlements by taking the distances between the centre of mass of each settlement patch and the centre of mass of its closest patch, then averaging these distances and comparing this average distance to the average distance in a random distribution to determine whether the rural settlements are aggregated [23,24,25]. The calculation formula is as follows:

(2) $$ANN=\frac{{\bar{D}}_{0}}{{\bar{D}}_{e}}=\frac{\sum_{i=1}^{n}}{\sqrt{n/A}/2}=\frac{2\sqrt{\lambda }}{n}\overset{n}{\underset{i=1}{\sum }}d_{i}$$

#### 2.4.3. Spatial Autocorrelation Measurements

The global clustering test is used for global spatial distribution patterns of rural settlement sizes, i.e., high or low value aggregation [42], and is expressed as:

(3) $$G\left(d\right)=\frac{\sum_{i=1j=1}^{n}\sum_{ij}^{n}w_{ij}\left(d\right)x_{i}x_{j}}{\sum_{i=1}^{n}\sum_{j=1}^{n}x_{i}x_{j}}$$

(4) $$Z\left(G\right)=\left[G-E\left(G\right)\right]/\sqrt{var\left(G\right)}$$

where *w_ij_* is the spatial weight defined by the distance rule; *x_i_* denotes the variable value of region *i*; *x_j_* denotes the variable value of region *j*; *E(G)* denotes the expected value of *G(d)* and *var(G)* denotes the variance of *G(d)*. Based on the value of *Z(G)*, it can be judged whether *G(d)* meets the significance level and whether there is a positive or negative spatial correlation. When *G(d)* is positive and *Z(G)* is statistically significant, it indicates a high-value cluster of rural settlement patches in the region. When *G(d)* is negative and *Z(G)* is statistically significant, it indicates a low-value cluster of rural settlement patches in the region.

#### 2.4.4. Spatial Hotspot Detection Analysis

Spatial hotspot detection analysis is a test for the presence of significant high and low values in a given area, and can be used as a spatial visualisation to reveal ‘hotspots’ and ‘coldspots’. In this paper, we focus on the size distribution of rural settlements [40]. The formula is:

(5) $$G_{i}^{*}\left(d\right)=\frac{\sum_{j=1}^{n}w_{ij}\left(d\right)x_{j}}{\sum_{j=1}^{n}x_{j}}$$

where G_i_^*^(d) is normalised in the same way as in Equation (3) to obtain Z(G_i_^*^). If Z(G_i_^*^) is positive and statistically significant, it indicates that the values around *i* are higher and belong to the “hot spot zone”. Conversely, it belongs to the “cold spot zone”.

### 2.5. Simulation of Rural Settlement Projections

#### 2.5.1. The MCCA Model

Traditional metacellular automata (CA) models assume that each cell is a specific land use type at each time step, ignoring the common mixed land use structure in land use cells. Mixed cells, which consist of a combination of multiple land-use types in proportion to their coverage, can better represent continuous land-use change and provide a new perspective for modelling the spatiotemporal dynamics of mixed land-use structures. MCCA (Mixed Cell CA) represents a new geospatial CA model for the spatiotemporal dynamics of mixed land-use structures. It provides a new approach to more dynamic mixed land use modelling away from static model analysis. One of the advantages of the MCCA model is its ability to simulate quantitative and continuous changes in multi-component cells, whereas pure cell CA models can only simulate qualitative and discrete changes in land use at the cell level. As a result, the MCCA model is able to simulate subtle changes in land use structure caused by small changes in socio-economic, ecological and political drivers, providing a detailed perspective for understanding land use change processes and improving simulation accuracy. The model first determines the total probability of change for a particular land use type and then simulates competition between land uses within the mixed study cell. When the land use component K wins the competition, the number of components K increases in the mixed plot, which also means that the other components decrease. Similarly, the probability of a decrease in the land use component K can be estimated from the probability of an increase in the other components. For a detailed explanation limited to space, please refer to related studies [42], where the MCCA-related equation is as follows.

(6) $$TP_{i,k}^{t}=DP_{i,k}\times \Omega_{i,k}^{t}\times Driv_{k}^{t}$$

where ${TP}_{i,k}^{t}$ is the total probability of change of land use component *k* of hybrid unit *i* at iteration *t*; $\Omega_{i,k}^{t}$ denotes the neighbourhood effect of hybrid unit *i*, which is the coverage of land use component *k* in the neighbourhood (the coverage of all components represents the land use structure of the neighbourhood).

(7) $${Driv}_{k}^{’t}=\{\begin{array}{ll} Driv_{k}^{t-1} & \;if\;\left|D_{k}^{t-1}\right|\leq \left|D_{k}^{t-2}\right|\\ Driv_{k}^{t-1}\times \frac{\left|D_{k}^{t-2}\right|+1}{\left|D_{k}^{t-1}\right|+1} & \;if\;0>D_{k}^{t-2}>D_{k}^{t-1}\\ Driv_{k}^{t-1}\times \frac{\left|D_{k}^{t-1}\right|+1}{\left|D_{k}^{t-2}\right|+1} & \;if\;D_{k}^{t-1}>D_{k}^{t-2}>0 \end{array}\;$$

where $Driv_{k}^{t-1}$ and $\left|D_{k}^{t-2}\right|$ denote the absolute value of the difference between the cumulative amount of land use type k and future demand at the *t-1st* and *t-2nd* iterations.

(8) $$\begin{array}{c} DA_{i,o}^{t}=\frac{SP_{i,o}^{t^{\prime }}}{\sum_{v=1}^{K-1}SP_{i,v}^{t^{\prime }}}\times IA_{i,k}^{t}v,o\neq k\\ \{\begin{array}{l} if\;con_{o\to k}=1\;\mathrm{then}\;IA_{i,k}^{t}=IA_{i,k}^{t};DA_{i,o}^{t}=DA_{i,0}^{t}\\ if\;con_{o\to k}=0\;\mathrm{then}\;IA_{i,k}^{t}=IA_{i,k}^{t}-DA_{i,0}^{t};DA_{i,o}^{t}=0 \end{array} \end{array}$$

where $DA_{i,o}^{t}$ denotes the proportional decrease in the land-use component *o* of hybrid unit *i* at iteration *t*; *con_o__→k_* denotes the conversion matrix that determines whether conversion of the original land-use type *o* to the target type *k* is allowed (1 indicates an unavoidable conversion, 0 indicates an impossible conversion). If conversion is not possible (*con_o__→k_* = 0), the value of $IA_{i,k}^{t}$ is adjusted to $IA_{i,k}^{t}-DA_{i,0}^{t},$ then the value of $DA_{i,o}^{t}ot$ is set to 0.

In this study, the MCCA model was used to simulate and predict the evolutionary trend of rural settlements in the Chaohu Peninsula area. Land use changes in the study area in 2015 were first simulated using 2000 as the base year, and the results were compared with the actual land use in 2015 to test the accuracy of the MCCA model in simulating land use changes in the Chaohu Peninsula.

#### 2.5.2. Different Developmental Scenario Settings

(1) Baseline scenario (BM, Benchmark) inertial development

The rule for the inertial development-based land use simulation of the Chaohu Peninsula is to calculate the rate of land use change between 2000 and 2015 so that it simulates the land use change from 2000 to 2015 at the same rate of change. The area of each land use type in 2030 was calculated using Markov chain, and the rate of change of each land use type from 2015–2030 is shown in Table 2.

(2) Urban–rural integration scenario (NTU, New-type urbanization)

This scenario focuses on analyzing the future land use and development of rural settlements after the implementation of the New-Type Urbanisation policy. The area of each land use type in 2030 has been revised according to the area of each land use type in the baseline scenario (Table 2). The implementation of the new urbanization policy will require the conversion of some rural settlements into land for urban construction.

(3) Tourism development scenario (TD)

This scenario mainly analyses the development direction of rural settlements on the Chaohu Peninsula in the context of the vigorous development of rural tourism. In recent years, the Hefei municipal government has vigorously promoted rural tourism in model villages and quality tourist villages. On this basis, the area of each land use type in 2030 is obtained by amending the area of each land use type under the BM scenario (Table 2).

#### 2.5.3. Land Use Simulation Drivers

**Figure 3:**
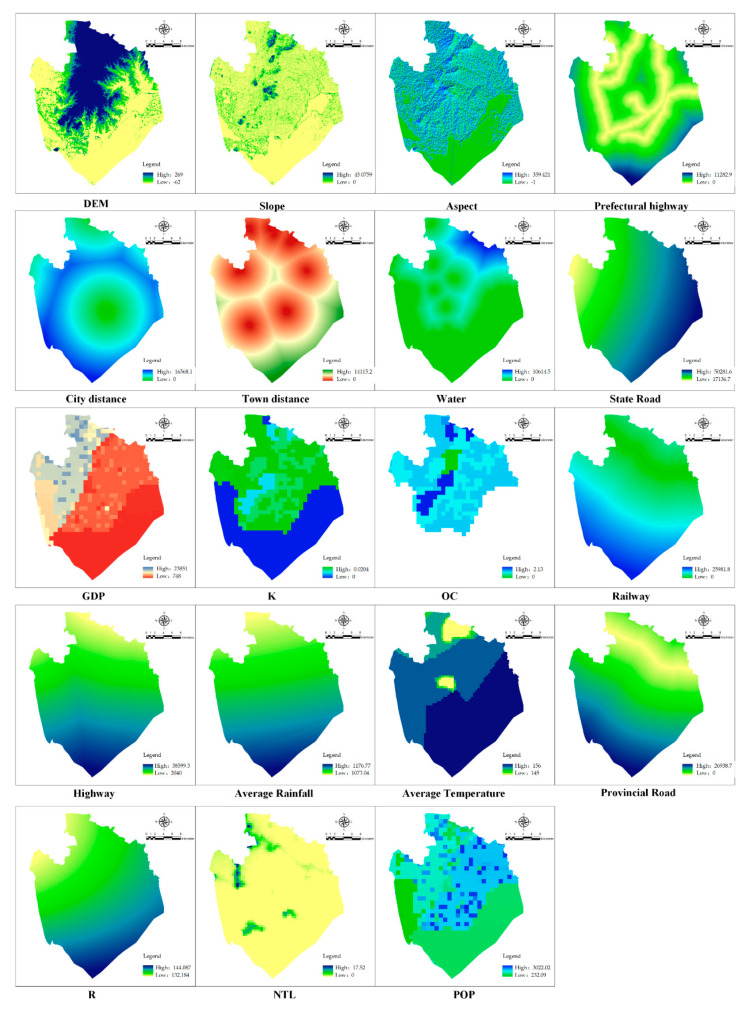
Driving factors for the evolution of rural settlements.

The suitability atlas obtained in the MCCA module is input using natural, social, and economic drivers to determine metacell transformation rules (Table 3) and simulate changes in land use patterns (Figure 3).

## 3. Results

### 3.1. Changes in the Size of Rural Settlements in the Chaohu Peninsula, 1980–2020

According to the results in Table 1, the total area of rural settlements on the Chaohu Peninsula increased continuously over the 40 years from 1980 to 2020, with a total increase of 8.05 km^2^. The growth rate of rural settlement area was not the same at different stages of development, with a growth rate of 0% between 1980 and 1990, 8% between 1990 and 2000, 4.57% between 2000 and 2010, and 2.97% between 2010 and 2020. Between 1980 and 2020, the number of rural settlement patches first increased and then decreased, with changes of 0%, 1.5%, −0.75%, and −0.75%, respectively.

**Figure 4:**
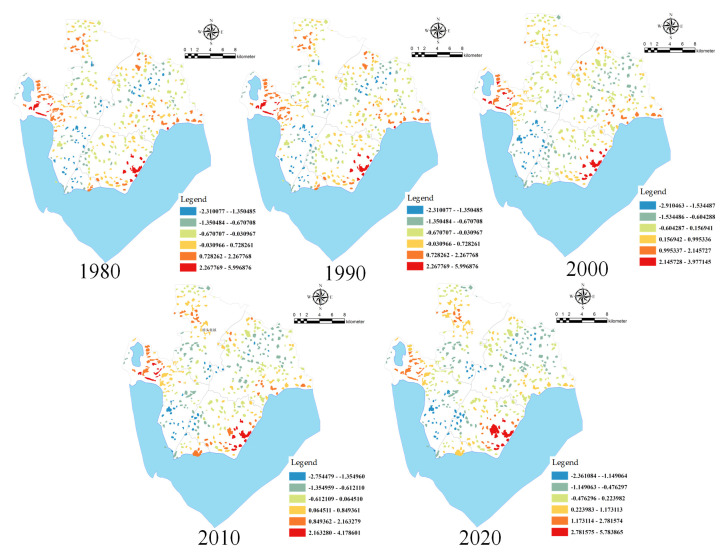
A “hotspot” map of the size of rural settlements on the Chaohu Peninsula.

The hotspot analysis tool was used to analyse the evolution of the size of rural settlements on the Chaohu Peninsula from 1980 to 2020 (Figure 4). During 1980–2020, the hotspots on the Chaohu Peninsula were mainly concentrated at the junction of Huanglu Town, Changlinhe Town, and Zhongmiao Street, whereas the cold spots were concentrated in Changlinhe Town near Hefei City and Huanglu Town near Chaohu Lake. The cold spots in Changlinhe Town showed a decreasing trend, while the cold spots in Huanglu Town showed an increasing trend. The number of hotspots over the past 40 years is 23. The number of hot spots in the past 40 years is 23, 23, 30, 30, and 27 respectively. The number of cold spots is 24, 24, 31, 21, and 17 respectively. The overall spatial distribution of rural settlements in the Chaohu Peninsula has the spatial characteristics of high-density, small-scale clustering distribution and low-density, large-scale random distribution.

### 3.2. Evolution of the Spatial Distribution of Rural Settlements in the Chaohu Peninsula

**Figure 5:**
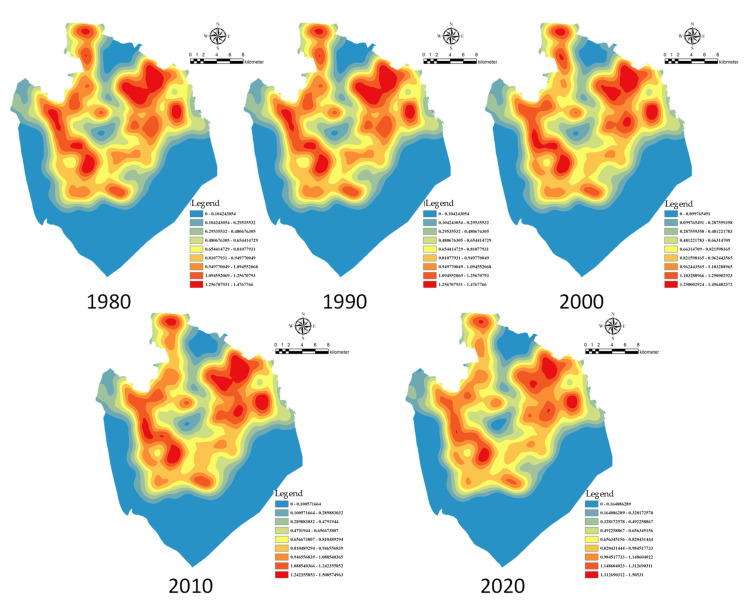
Evolution of the nuclear density of rural settlements in the Chaohu Peninsula.

The spatial distribution of rural settlements on the Chaohu Peninsula can be characterised by morphological characteristics and spatial distribution patterns (Figure 5). As can be seen from Table 4, during the period 1980–2020, the average patch shape index (SHAPE_MN) of rural settlements first decreases and then increases, and the landscape shape index (LSI) and maximum patch index (LPI) continue to increase, reflecting that the patch shape of rural settlements on the Chaohu Peninsula tends to be complex and the development of rural settlements has a tendency to concentrate. The spatial expansion of rural settlements is obvious, and the area of land for construction per capita has increased from 150 m^2^ to 220 m^2^, reducing the degree of land use intensification.

Analysis of the nuclear density analysis map of rural settlements from 1980 to 2020 shows that: (1) the density of rural settlements in all five years shows a spatial pattern of “dense in the northwest and sparse in the southeast”, and the overall distribution is relatively scattered; (2) the density of rural settlements in the study area near Hefei and Chaohu city gradually decreased over the 40 years; (3) The average distances (m) of rural settlements during the 40 years were 682.58, 682.58, 695.56, 692.82, and 691.50, and the ANN values were 1.03, 1.03, 1.06, 1.05, and 1.04, respectively, with the overall distance of rural settlements gradually increasing and the overall density decreasing. The scattered distribution of rural settlements increases from 1980 to 2020 and decreases from 2000 to 2020, which is related to the urban development around the study area and related rural development policies.

**Figure 6:**
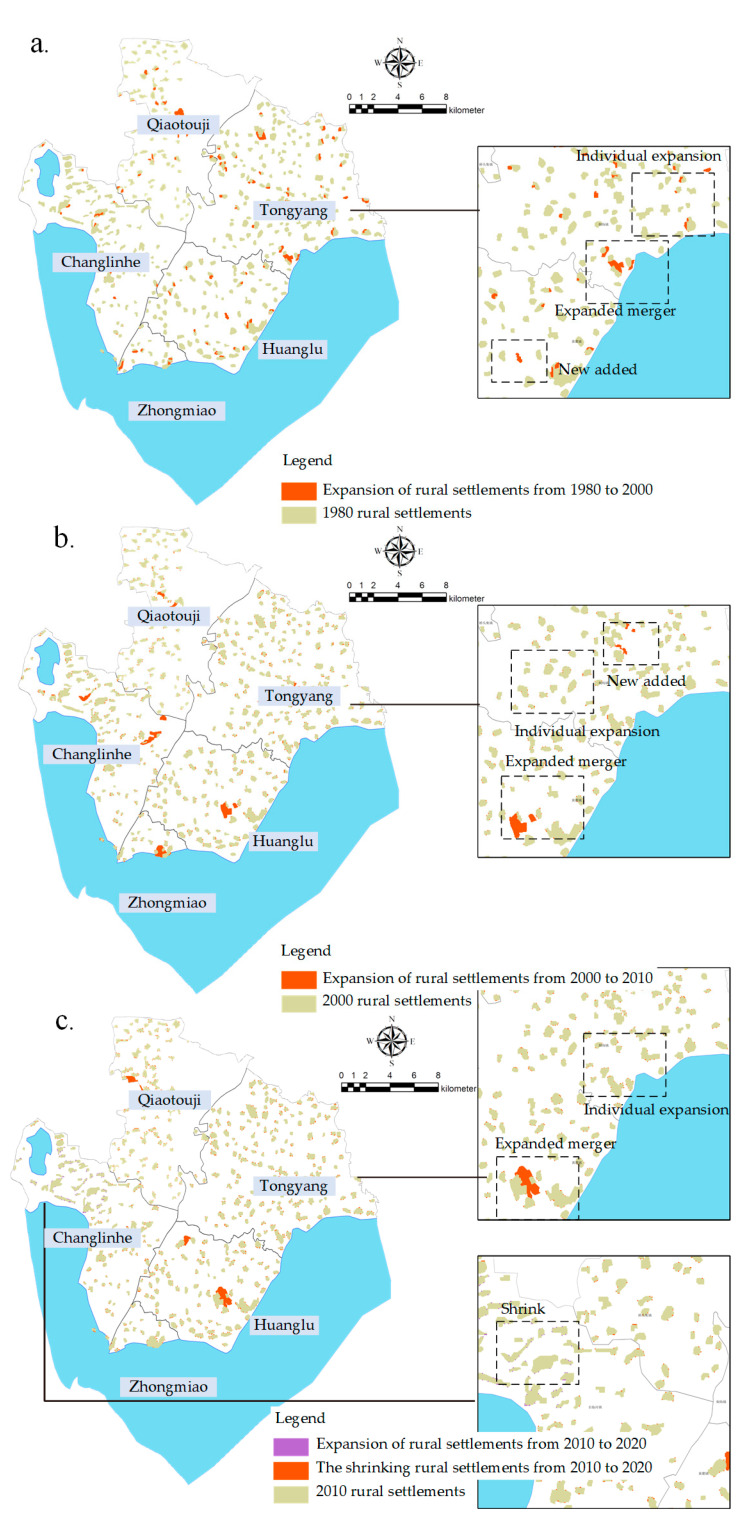
The evolution model of rural settlements in the suburbs of Hefei from 1980 to 2020. (**a**) Change pattern of residential areas in the study area from 1980 to 2000, (**b**) Change pattern of residential areas in the study area from 2000 to 2010, (**c**) Change pattern of residential areas in the study area from 2010 to 2020.

During 1980–2020, the spatial pattern of rural settlements in the Chaohu Peninsula can be divided into four patterns (Figure 6): expansion outwards from the original settlements (individual expansion); spontaneous merging of small and scattered rural settlements into larger settlements (combined expansion); shrinkage of rural settlements (reduction); and addition of rural settlements (new addition). The spatial evolution of rural settlements from 1980 to 2000 was mainly based on the expansion pattern (Figure 6a), during which a large number of rural settlements in the study area expanded in size, and individual and combined expansions were widely seen in the study area, with new areas being added mainly in the Qiaotou township and Huanglu township areas. The expansion pattern of the 1980s and 2000s continues (Figure 6b), but the overall expansion decreases, and there is still extensive consolidation of rural settlements in Changlinhe Town, Zhongmiao Street Road, and Huanglu Town, which is more related to real estate development. In 2010–2020 (Figure 6c), a large number of rural settlements decreased in size in Changlinhe Town near the Hefei city area. Whereas no new rural settlements are built throughout the study area as well as in Huanglu Town and Zhongmiao Street Road, merging and expanding rural settlements still occur.

### 3.3. Simulation of Rural Settlement Projections for the Chaohu Peninsula under Different Development Scenarios

#### 3.3.1. Verification of the Accuracy of the Simulation Results

Land use changes from 2000–2015 were first simulated and the accuracy of the simulation was verified based on the Kappa and mc FOM coefficients. The accuracy of the predictions for arable land, forest land, grassland, water, wetland, towns, and rural settlements were 0.97, 0.99, 0.97, 0.99, 0.98, 0.81, and 0.87, respectively, with the accuracy of the simulation being higher than 0.8. The consistency of the simulation results with the land use in 2020 was very good. The accuracy of the simulation is higher than 0.8, and the consistency between the simulation results and the land use in 2020 is very good.

#### 3.3.2. Modelling the Evolution of Rural Settlements under Different Scenarios

After the accuracy met the research needs, the MCCA was used to simulate the land use status of the Chaohu Peninsula in 2030 and to calculate the conversion between land types under different development scenarios from 2015 to 2030 using the land use transfer matrix, which can reflect the amount and direction of regional land use conversion.

(1) Baseline scenario (BM, Benchmark)

**Figure 7:**
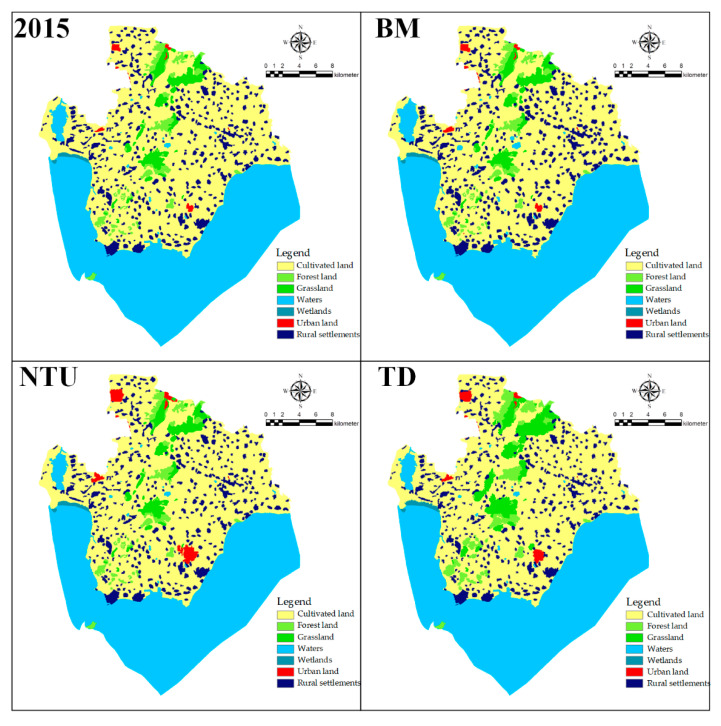
Simulation of different scenarios of land use in the Chaohu Peninsula.

Under the BM scenario (Figure 7 and Table 5), the area of rural settlements and urban land will increase by 1.1% and 0.9% respectively, while the area of arable land, forest land and grassland will decrease by 2%, 0.01% and 0.01% respectively. Moreover, 2020–2030 will see the expansion of rural settlements mainly in their own outward direction, in addition to an increase in the area of water. The expansion of construction land in Huanglu township and Qiaotou township is obvious, mainly because of the proximity to the main traffic routes, which easily attracts population concentration. However, the continuous decline of arable land, forest land, and grassland area is not conducive to future development. It can be observed that with the continuous promotion of urbanization, the expansion of construction land in the Chaohu Peninsula is more obvious under natural development and rapid development scenarios, and land resources are facing greater unsustainability.

(2) Urban–rural integration scenario (NTU, New-Type Urbanization)

Under the NTU scenario, it can be observed from Figure 7 and Table 6 that there is a large increase in urban land, with urban land increasing from 0.2% to 1.02% and rural settlements shrinking to some extent throughout the Chaohu Peninsula. The rural settlement sites decreased by 0.2%. This includes a decrease in the rate of reduction of arable land compared to the BM scenario, which decreased by 1.4%. According to the land use transfer matrix of the NTU scenario, the main reason for the expansion of the town limits is the annexation of surrounding rural settlements and arable land.

(3) Tourism development scenario (TD)

Under the TD scenario (Figure 7 and Table 7), the area of towns and rural settlements on the Chaohu Peninsula decreases to 7.82%, urban land is expanded to some extent, woodlands, grasslands, and wetlands all increase to some extent, and the overall ecological environment of the study area rises. In the TD scenario, the shrinkage of arable land is greater than in the NTU scenario, which may be related to the actual development aspirations of the Chaohu Peninsula. As a large amount of arable land is abandoned and local farmers are not very motivated to cultivate land, in order to develop tourism, some of the arable land is transformed into forest land, grassland, and other tourist land with ornamental and economic value, while ensuring that permanent basic protected farmland is not encroached upon.

## 4. Discussion

### 4.1. Factors of the Changing Scale of Rural Settlements in the Chaohu Peninsula

During the period from 1980 to 2020, the scale of change of rural settlements in the Chaohu Peninsula continued to grow, mainly due to the continuous growth of the population and the rapid economic development of the Chaohu Peninsula. After the reform and opening up, the government and the market broke the restrictions of the original production relations, farmers became more motivated to farm, and the gross agricultural product of the four townships in the study area continued to grow, contributing to the expansion of rural settlements.

The changes in the distribution of cold hotspots in the distribution of the size of rural settlements in different periods on the Chaohu Peninsula are closely related to the economic development of the townships in the district, the distribution of industries and the distribution of road networks throughout the district. From 1980 to 2020, the hotspots of rural settlements were always concentrated in areas with better geographical locations, larger populations, and more natural resources, such as the lakefront areas of Huanglu Town and Zhongmiao Street, as well as areas close to parts of the Hefei city area. As the Chaohu peninsula has promoted agricultural and industrial development, the population and economic levels of towns with consolidated arable land and developed industries have risen sharply, which in turn has led to the expansion of rural settlements in these areas and the formation of concentrated hotspots. At the same time, the Chaohu Peninsula itself is rich in natural beauty and tourism resources, and the scale of rural settlements in tourist development zones has increased significantly, creating more hotspots.

### 4.2. Driving Forces behind the Evolution of the Rural Settlement Pattern on the Chaohu Peninsula

The distribution pattern of rural settlements on the Chaohu Peninsula has been more randomly distributed across the study area since reform and opening up, but there has been a gradual increase in the concentration of rural settlements. There are two possible reasons for this characteristic. On the one hand, the overall spatial pattern of rural settlements on the Chaohu Peninsula is “dense in the north-west and sparse in the south-east”, and this distribution is influenced by the topographic relief. On the other hand, the level of urbanisation and economy on the Chaohu Peninsula is low, and the evolution of rural settlements relies mainly on the traditional agricultural development model. In addition, the main land type in the study area is arable land, and farmers are more inclined to build houses near arable land due to the influence of the traditional Chinese agricultural land system [20,32]. The random distribution of arable land has led to a pattern of random distribution of rural settlements.

### 4.3. Simulation and Prediction Analysis of Rural Settlements on the Chaohu Peninsula

The future development trend of the rural settlements on the Chaohu Peninsula depends on the development scenario adopted. Under the BM scenario, the change in land use types follows the same development pattern as in 2010–2015. Urbanization will convert rural settlements around cities into urban sites. At the same time, villagers will seek a more spacious and comfortable living environment due to rising living standards, which will lead to the construction of a large number of new rural settlements on cultivated land.

Under the NTU scenario, as the Chaohu peninsula is close to the Hefei and Chaohu urban areas, its construction and development will need to be implemented in strict accordance with the NTU plan in order to strengthen the regional interface, and more employment opportunities will attract farmers from the surrounding areas to the urban areas. Therefore, in this scenario, the size of rural settlements will be reduced, and the area of urban land and other construction land will increase.

Under the TD scenario, the Chaohu Peninsula will become an important tourist destination and rural tourism will flourish due to the innate geographical advantage of Chaohu and the fact that it is the core of the Chaohu National Tourism and Recreation Demonstration Zone. In addition, according to the Chaohu Peninsula plan, the scale of its tourism industry is expected to further expand. Rural tourism in the region will lead to a significant increase in the number and area of “special farmhouses” in and around the scenic area, leading to an increase in the size of rural settlements, but a decrease in the total number to meet the actual needs of town development.

### 4.4. Policy Recommendations

After the reform and opening up, due to the large-scale construction of the Chaohu Peninsula, the original structure of rural settlements was destroyed, and their external form became irregular. Therefore, it is necessary to strengthen the unified planning of rural housing construction, and scientifically plan the scale and layout of rural settlements. The new urbanization policy has facilitated a shift in population from rural to urban areas. As a result, a reduction in the size of the rural population may lead to a reduction in the number of rural settlements. Therefore, the government should consider the management and control of the registered rural population and allocate rural settlements of an appropriate size to meet the needs of these populations.

Conversely, the large number of farmhouses established as a result of the development of rural tourism in the Chaohu Peninsula will increase the size of rural settlements in some areas, in order to prevent conflict with urban and rural policies. Therefore, the higher government should plan the layout and scale of rural settlements according to the main development patterns of different townships, in order to control the direction of transformation of rural settlements under different development patterns. For example, the mountainous areas in the northern part of the Chaohu District should focus on developing tourism and creating high-quality tourist villages and towns. The western part of the Chaohu Peninsula should focus on the development of urban industries to further promote urbanization and integrate with the urban development of Hefei.

## 5. Conclusions

This paper uses land use data from 1980 to 2020 as a research data source to obtain historical data about rural settlements on the Chaohu Peninsula and to analyse the evolution of the spatial pattern of rural settlements over a long time span. By setting different land-use development scenarios, the future evolutionary trends of rural settlements in the study area are predicted. The following conclusions were drawn.

(1) The spatial pattern evolution characteristics of rural settlements since the reform and opening up reflect the level of economic development and the interaction between policies and human activities in different periods. The spatial distribution pattern of rural settlements on the Chaohu Peninsula over the past 40 years has been characterised by high-density small-scale distribution and low-density large-scale agglomeration distribution.

(2) Over the past 40 years, the degree of intensification of rural settlements on the Chaohu Peninsula has been low, with the density of rural settlements showing a spatial pattern of “dense in the northwest and sparse in the southeast”, and the overall distribution has been relatively scattered.

(3) The transformation of rural settlements into urban land is the main reason for the decrease in the number of rural settlements on the Chaohu Peninsula. The rapid increase in rural population and economic growth is the main reason for the increasing size of rural settlements. The implementation of relevant development plans is an important reason for the continuous expansion of the spatial structure of rural settlements. The continuation of the rural land system and changes in family structure have also influenced the evolution of rural settlements.

In using the MCCA model to predict land use development on the Chaohu Peninsula, we did not consider policies regarding the city of Hefei, as it was difficult to quantify these policies when running the model. As the new urbanization development plan and tourism development plan for the Chaohu peninsula do not specify the amount of land use in 2030, the size of each land use category is mainly calculated based on the relevant government documents that have been published and the future population size projections. In this way, there may be a subjective element in the land indicator allocation process. However, as the allocation of each land type is determined after in-depth surveys of the local area and thorough validation of the data, the land use quantity projections can largely reflect the future development trends of the Chaohu Peninsula. In addition, the evolution of the rural settlement driver effect from 2020 to 2030 is likely to change. These changes in the driving forces cannot be accurately predicted and considered in the simulations at present, limited by the availability of data. Methods to address this issue will be considered in future studies.

## Figures and Tables

**Table 1:** Description table of landscape pattern index.

Index	Formula	Meaning	Parameter Description
Patch area	$CA=\overset{n}{\underset{i=1}{\sum }}a_{i}$	The total area of patches in the study area.	n is the number of patches; $a_{i}$ is the area of the i-th patch.
Number of patches	NP=n	The total number of patches in the study area.	n is the number of patches.
Maximum patch index	LPI= $\frac{MAX\left(a_{1},\ldots ,a_{3}\right)}{A}$	Reflects the degree of influence of the largest patch on the entire rural residential area, the larger the value, the higher the degree of patch land agglomeration.	$a_{n}$ is the largest patch area.
Landscape shape index	$LSI=\frac{0.25E}{\surd A}$	Reflects the complexity of the patch, the larger the value, the more irregular the shape of the patch and the greater the tortuosity of the boundary.	E is the total length of all patches in the landscape.
Mean patch shape index	$\begin{array}{c} \mathrm{SHAPE}\text{\_}\mathrm{MN}\;=\;\frac{\sum_{i=1}^{m}\sum_{j=1}^{n}\left(\frac{0.25c_{ij}}{\sqrt{a_{ij}}}\right)}{N} \end{array}$	Reflects the complexity of the external shape of the patch.	$c_{ij}$ is the perimeter of the patch, $a_{ij}$ is the area of the patch.
Mean patch area	$MPS=\frac{CA}{n_{i}}$	Reflects the degree of fragmentation of the patch.	CA is the total area of patches; $n_{i}$ is the total number of patches.

**Table 2:** Area ratio of different land use types in Chaohu Peninsula in 2000 and 2015 and the predicted area ratio in 2030 under different scenarios (%).

Year	2000	2015	2030		
			BM	NTU	TD
Farmland	47.8	46.0	44.0	44.6	43.8
Forest	2.63	2.62	2.61	2.61	3.56
Grassland	2.9	2.84	2.83	2.83	4.27
Water body	39.0	39.6	40.5	39.6	39.6
Wetland	0.24	0.24	0.24	0.24	0.32
Urban land	0.2	0.32	0.41	1.02	0.63
Rural settlement	7.23	8.30	9.41	8.10	7.82

**Table 3:** Driving factor description table.

Type	File Name	Data Description
Land use data	Land use in 2010	Land use types are farmland, forest, grassland, water body, wetland, urban land, rural settlement
	Land use in 2020	
Restrict conversion of land	Open water	River water system/land policy data
Data on Driving Forces of Land Use Change	Dem	Elevation
	Slope	slope
	Aspect	Aspect
	Railway	Distance to railway
	State Road	Distance to national highway
	Highway	Distance to highway
	Provincial Road	Distance to provincial highway
	Prefectural highway	County road
	Average Rainfall	Mean rainfall
	Average Temperature	Mean temperature
	OC	Organic matter content
	K	Soil erodibility factor
	City distance	Distance to the city
	R	Rainfall erosivity factor
	Town distance	Distance to town
	GDP	Gross national product
	POP	population
	Water	Distance to water source
	NTL	Night light data

**Table 4:** Changes in the landscape pattern index of rural residential areas from 1980 to 2020.

Year	1980	1990	2000	2010	2020
NP	394	394	400	397	394
CA	4909.86	4909.86	5306.40	5549.04	5714.1
SHAPE_MN	1.2478	1.2478	1.2372	1.2470	1.2536
MPS	12.46	12.46	13.27	14.00	14.50
LPI	0.1521	0.1521	0.1667	0.1671	0.2601
LSI	24.7479	24.7479	24.5267	24.6318	24.5992

**Table 5:** Land use transfer matrix under BM scenario (km^2^).

Type	Farmland	Forest	Grassland	Water Body	Wetland	Urban Land	Rural Settlement
Farmland	329.026	0.00	0.122	4.155	0.079	0.621	7.918
Forest	0.00	19.56	0.00	0.0009	0.00	0.00	0.011
Grassland	0.162	0.00	20.940	0.00	0.00	0.00	0.076
Water body	0.022	0.00	0.00	294.37	0.00	0.00	0.004
Wetland	0.019	0.00	0.00	0.00	1.796	0.00	0.001
Urban land	0.008	0.00	0.00	0.00	0.00	2.43	0.00
Rural settlement	0.00	0.00	0.00	0.00	0.00	0.00	61.51

**Table 6:** Land use transfer matrix under NTU scenario (km^2^).

Type	Farmland	Forest	Grassland	Water Body	Wetland	Urban Land	Rural Settlement
Farmland	335.152	0.353	0.215	0.277	0.052	4.130	1.742
Forest	0.070	19.317	0.054	0.0369	0.00	0.0981	0.00
Grassland	0.074	0.056	20.747	0.000	0.00	0.306	0.00
Water body	0.233	0.014	0.0000	294.082	0.049	0.00	0.014
Wetland	0.0009	0.0000	0.00	0.00	1.814	0.00	0.00
Urban land	0.019	0.0000	0.00	0.00	0.00	2.4192	0.00
Rural settlement	2.030	0.011	0.016	0.040	0.00	0.624	58.813

**Table 7:** Land use transfer matrix under TD scenario (km^2^).

Type	Farmland	Forest	Grassland	Water Body	Wetland	Urban Land	Rural Settlement
Farmland	318.97	6.9165	10.539	0.0288	0.5625	2.1465	2.758
Forest	0.126	18.542	0.869	0.00	0.00	0.0369	0.0018
Grassland	0.088	0.8766	20.073	0.00	0.00	0.1341	0.0009
Water body	0.2457	0.00	0.00	294.134	0.00	0.00	0.0117
Wetland	0.00	0.00	0.00	0.00	1.814	0.00	0.00
Urban land	0.059	0.004	0.058	0.00	0.00	2.3166	0.0009
Rural settlement	5.910	0.107	0.183	0.002	0.00	0.0108	55.318

## Data Availability

Data sharing is not applicable to this article.
